# Phenotypic and genotypic characterisation of multiple antibiotic-resistant *Staphylococcus aureus* exposed to subinhibitory levels of oxacillin and levofloxacin

**DOI:** 10.1186/s12866-016-0791-7

**Published:** 2016-07-29

**Authors:** Ara Jo, Juhee Ahn

**Affiliations:** 1 Department of Medical Biomaterials Engineering, Kangwon National University, Chuncheon, Gangwon 24341 Republic of Korea; 2 Institute of Bioscience and Biotechnology, Kangwon National University, Chuncheon, Gangwon 24341 Republic of Korea

**Keywords:** Lactamase, Biofilm, Efflux pump, Antibiotic resistance, *Staphylococcus aureus*, Gene expression, Levofloxacin, Oxacillin

## Abstract

**Background:**

The emergence and spread of multidrug resistant methicillin-resistant *Staphylococcus aureus* (MDR-MRSA) has serious health consequences in the presence of sub-MIC antibiotics. Therefore, this study was designed to evaluate β-lactamase activity, efflux activity, biofilm formation, and gene expression pattern in *Staphylococcus aureus* KACC 10778, *S. aureus* ATCC 15564, and *S. aureus* CCARM 3080 exposed to sublethal concentrations of levofloxacin and oxacillin.

**Results:**

The decreased MICs were observed in *S. aureus* KACC and *S. aureus* ATCC when exposed to levofloxacin and oxacillin, while and *S. aureus* CCARM remained resistance to streptomycin (512 μg/mL) in the presence of levofloxacin and imipenem (>512 μg/mL) in the presence of oxacillin. The considerable increase in extracellular and membrane-bound β-lactamase activities was observed in *S. aureus* ATCC exposed to oxacillin (>26 μmol/min/mL). The antibiotic susceptibility of all strains exposed to EPIs (CCCP and PAβN) varied depending on the classes of antibiotics. The relative expression levels of adhesion-related genes (*clfA*, *clfB*, *fnbA*, *fnnB*, and *icaD*), efflux-related genes (*norB*, *norC*, and *qacA/B*), and enterotoxin gene (*sec*) were increased more than 5-fold in *S. aureus* CCARM. The *eno* and *qacA/B* genes were highly overexpressed by more than 12- and 9-folds, respectively, in *S. aureus* CCARM exposed to levofloxacin. The antibiotic susceptibility, lactamase activity, biofilm-forming ability, efflux activity, and gene expression pattern varied with the intrinsic antibiotic resistance of *S. aureus* KACC, *S. aureus* ATCC, and *S. aureus* CCARM exposed to levofloxacin and oxacillin.

**Conclusions:**

This study would provide useful information for better understating of combination therapy related to antibiotic resistance mechanisms and open the door for designing effective antibiotic treatment protocols to prevent excessive use of antibiotics in clinical practice.

**Electronic supplementary material:**

The online version of this article (doi:10.1186/s12866-016-0791-7) contains supplementary material, which is available to authorized users.

## Background

Over the last several decades, the overuse and misuse of broad-spectrum antibiotics has contributed to the increased emergence of antibiotic resistant pathogens such as methicillin-resistant *Staphylococcus aureus* (MRSA) [1, 2]. MRSA infections can cause mild to severe diseases, including skin lesion, toxic shock syndrome, endocarditis, osteomyelitis, and meningitis [2, 3]. Both hospital-acquired MRSA and community-acquired MRSA have currently become the leading causes of morbidity and mortality worldwide [3, 4]. Furthermore, MRSA can develop co-resistance to different classes of antibiotics, including fluoroquinolones, aminoglycosides, macrolides, tetracyclines, and β-lactams, known as multidrug resistant (MDR) MRSA [5–7]. The MDR-MRSA can frequently be exposed to subinhibitory concentrations of antibiotics, which leads to gene transfer, biofilm formation, and virulence gene expression [1]. The emergence and spread of MDR-MRSA has serious health consequences in the presence of sub-MIC antibiotics. Therefore, the effective control of MDR-MRSA is a research priority in hospitals and other healthcare facilities.

The different classes of antibiotics are used to improve the treatment of MDR bacterial infections, specifically carbapenem-resistant Enterobacteriaceae (CRE), which is known as combination therapy [8]. The benefits of using the combination therapy include the extension of antibiotic spectrum, synergistic enhancement of antibiotic activity, and decrease in the frequency of antibiotic resistance [8, 9]. Compared to the mono-therapy, the combination therapy can reduce the excessive use of antibiotics. However, controversially, there are also risks associated with the combination therapy. The selection of antibiotic resistance varies depending on the concentrations exposed to antibiotics [10]. Bacteria exposed to sublethal concentrations are likely to have a wide range of mutation variance compared to those exposed to lethal concentrations of antibiotics [1]. Relatively, few studies have investigated the mechanisms of resistance in MRSA under combination therapy. Therefore, in this study, we evaluated the physiological and molecular responses of MRSA to different classes of antibiotics in the presence of oxacillin and levofloxacin as measured by β-lactamase activity, efflux activity, biofilm formation, and gene expression pattern.

## Methods

### Bacterial strains and culture conditions

Strains of *S. aureus* KACC 10778, *S. aureus* ATCC 15564, and *S. aureus* CCARM 3080 were obtained from American Type Culture Collection (ATCC, Manassas, VA), Korean Agricultural Culture Collection (KACC, Suwon, Korea), and Culture Collection of Antibiotic Resistant Microbes (CCARM, Seoul, Korea), respectively. All strains were cultured in tryptic soy broth (TSB; BD, Becton, Dickinson and Co., Sparks, MD) at 37 °C for 20 h. After cultivation, cultures were centrifuged at 3000 × g for 20 min at 4 °C, washed twice with phosphate-buffered saline (PBS, pH 7.2), and then used for assays.

### Single antibiotic susceptibility assay

The susceptibility of *S. aureus* KACC 10778, *S. aureus* ATCC 15564, and *S. aureus* CCARM 3080 to each antibiotic listed in Additional file 1: Table S1 was evaluated according to the Clinical Laboratory Standards Institute (CLSI) procedure with minor modification [11]. All antibiotic stock solutions were prepared by dissolving in distilled water (ampicillin, cefoxitin, ceftazidime, ceftriaxone, gentamicin, meropenem, oxacillin, streptomycin, and vancomycin), ethaol (chloramphenicol and tetracycline), glacial acetic acid (ciprofloxacin, levofloxacin, and norfloxacin), dimethyl sulfoxide (DMSO; imipenem) to obtain a final concentration of 10.24 mg/mL. Each stock solution (100 μL) was serially (1:2) diluted from 512 μg/mL with TSB in 96-well microtiter plates (BD Falcon, San Jose, CA). All strains were inoculated at a level of 10^6^ CFU/mL and incubated at 37 °C for 18 h. Minimum inhibitory concentration (MIC) was determined at the lowest concentration of each antibiotic at which there is no visible growth. MIC breakpoints were used to define susceptible (S), intermediate (I), and resistant (R) strains [12, 13].

### Combination antibiotic sensitivity test

The susceptibility of *S. aureus* KACC 10778, *S. aureus* ATCC 15564, and *S. aureus* CCARM 3080 to each antibiotic was also evaluated in the presence of oxacillin or levofloxacin. All strains (10^5^ CFU/mL each) were inoculated in 96-well microtiter plates containing serial (1:2) antibiotic dilutions and basal antibiotic (oxacillin or levofloxacin; 1/2 MIC). MICs were determined as above mentioned.

### β-lactamase activity assay

The ability of β-lactamase to hydrolyze nitrocefin was evaluated by using a spectrophotometric assay with minor modifications [14]. *S. aureus* KACC 10778, *S. aureus* ATCC 15564, and *S. aureus* CCARM 3080 cells exposed to 1/2 MIC of oxacillin or levofloxacin at 37 °C for 20 h were centrifuged at 3000 × g for 20 min at 4 °C. The cells suspended in PBS and cell-free supernatants were mixed with 20 μL of 1.5 mM nitrocefin and incubated at 37 °C for 30 min. The absorbance was measured every 5 min at 515 nm [15].

### Ethidium Bromide (EtBr) cartwheel method

The cultured strains were centrifuged and then rinsed with PBS. The harvested cells were suspended in PBS with and without EPIs (CCCP, 0.5 μg/mL; PAβN, 8 μg/mL) [16, 17]. TSA plates containing EtBr (1 μg/mL) were prepared under darkness and divided into 9 sectors with cartwheel pattern. The prepared cells were swabbed on EtBr-agar plates and then incubated at 37 °C for 16 h. After incubation, the swabbed EtBr-agar plates were observed under UV illumination (Gel-doc XR System; Bio-Rad, Hertfordshire, UK).

### Competitive efflux pump inhibition assay

The efflux pump activity of *S. aureus* KACC 10778, *S. aureus* ATCC 15564, and *S. aureus* CCARM 3080 was evaluated in the absence and presence of efflux pump inhibitors (EPIs), carbonyl cyanide-*m*-chlorophenyl hydrazone (CCCP) and phenylalanine-arginine-β-naphthylamide (PAβN). The changes in antibiotic susceptibility of *S. aureus* ATCC 15564, *S. aureus* KACC 10778, and *S. aureus* CCARM 3080 exposed to EPIs were determined as above mentioned.

### Biofilm-forming ability assay

The biofilm formation potential by *S. aureus* KACC 10778, *S. aureus* ATCC 15564, and *S. aureus* CCARM 3080 was evaluated in the absence and presence of oxacillin or levofloxacin, which was based on the ability of strains to attach on 12-well polystyrene microtiter plate surface. All strains (10^6^ CFU/mL each) were inoculated in TSB containing 1/2 MIC of oxacillin or levofloxacin and incubated at 37 °C for 24 h. After cultivation, each well was gently washed with PBS to remove loosely adhered cells. The adhered cells were harvested by using a cell scraper (Thermo Scientific Nunc, Rochester, NY). The collected cells were dispersed in PBS (1 mL) and then serially diluted (1:10) with PBS. The proper dilutions were plated on trypticase soy agar (TSA) using an Autoplate Spiral Plating System (Spiral Biotech Inc., Norwood, MA, USA). The plates were incubated at 37 °C for 24–48 h to enumerate adhered cells using a QCount Colony Counter (Spiral Biotech Inc.).

### Quantitative PCR assay

The RNA extraction was carried out using RNeasy Protect Bacteria Mini kit (Qiagen, Hilden, Germany). Briefly, *S. aureus* KACC 10778, *S. aureus* ATCC 15564, and *S. aureus* CCARM 3080 cells (0.5 mL each) exposed to 1/2 MIC of oxacillin or levofloxacin at 37 °C for 20 h were mixed with 1 ml of RNAprotect Bacteria Reagent. The mixtures were centrifuged at 5000 × *g* for 10 min, and the collected cells were lysed with a buffer containing lysozyme. The lysates were mixed with ethanol to extract RNA using an RNeasy mini column. In order to synthesize cDNA, the RNA extracts were rinsed with a Wipe buffer to remove the genomic DNA and then mixed a master mixture of reverse transcriptase, RT buffer, and RT primer mix and then incubated at 42 °C for 15 min followed by 95 °C for 3 min. The PCR mixture (20 μl) containing 10 μl of 2× QuantiTect SYBR Green PCR Master, 2 μl of each primer, and 2 μl of cDNA, and 4 μl of RNase-free water was denatured at 95 °C for 30 s, followed by 45 cycles of 95 °C for 5 s, 55 °C for 20 s, and 72 °C for 15 s using an iCycler iQ™ system (Bio-Rad Laboratories, Hemel Hempstead, UK). The custom-synthesized oligonucleotides using IDT (Integrated DNA Technologies Inc., Coralville, IA, USA) as primers of *S. aureus* are listed in Additional file 2: Table S2. The relative gene expression levels were estimated using the comparative method [18]. The C_T_ values of target genes in *S. aureus* KACC 10778, *S. aureus* ATCC 15564, and *S. aureus* CCARM 3080 cells exposed to 1/2 MIC of oxacillin or levofloxacin were compared to the C_T_ values obtained from the control cells, respectively. The reference gene (16S ribosomal RNA) was used for normalization of target gene expression.

### Statistical analysis

Data were analyzed by the Statistical Analysis System (SAS) software. All analyses were carried out in duplicate for three replicates. The general linear model (GLM) and Fisher’s least significant difference (LSD) procedures were used to determine significant mean differences at *p* < 0.05.

## Results

### Antibiotic susceptibility of *S. aureus*

The MICs of selected antibiotic against *S. aureus* KACC 10778, *S. aureus* ATCC 15564, and *S. aureus* CCARM 3080 were determined in absence and presence of antibiotics (levofloxacin and oxacillin) (Table 1). *S. aureus* KACC 10778, *S. aureus* ATCC 15564, and *S. aureus* CCARM 3080 were classified on the basis of MIC breakpoints as antibiotic-sensitive, intermediate, and antibiotic-resistant strains, respectively. *S. aureus* KACC 10778 and *S. aureus* ATCC 15564 were relatively sensitive to most antibiotics when compared to *S. aureus* CCARM 3080 which was resistant to all antibiotics with the exception of chloroamphenicol and vancomycin. The MIC values of most antibiotics against all strains tested were decreased in the presence of levofloxacin and oxacillin. However, no changes were observed in susceptibilities of *S. aureus* KACC 10778 to chloramphenicol when exposed to levofloxacin, gentamicin exposed to oxacillin, tetracycline exposed to levofloxacin and oxacillin and *S. aureus* ATCC 15564 to ceftazidime exposed to levofloxacin and tetracycline exposed to levofloxacin and oxacillin. The reduced susceptibility of *S. aureus* CCARM 3080 to streptomycin was observed in the presence of levofloxacin.

**Table 1:** MIC (μg/mL) of selected antibiotics against *Staphylococcus aureus* in a half MIC of levofloxacin (LVX) or oxacillin (OXA)

Antibiotic	*S. aureus* KACC 10778			*S. aureus* ATCC 15564			*S. aureus* CCARM 3080		
	Control	LVX	OXA	Control	LVX	OXA	Control	LVX	OXA
Ampicillin	0.25(S)	0.125	0.25	32(R)	8	4	256(R)	<1	16
Cefoxitin	2(S)	0.5	0.5	4(S)	1	0.25	>512(R)	<1	32
Ceftazidime	8(R)	2	4	16(R)	16	4	512(R)	4	128
Ceftriaxone	2(R)	1	1	4(R)	1	0.25	>512(R)	<2	512
Chloramphenicol	8(S)	8	4	8(S)	4	4	16(I)	4	8
Ciprofloxacin	0.5(S)	0.125	0.25	0.5(S)	0.25	0.125	32(R)	1	8
Gentamicin	1(S)	0.5	1	16(R)	2	1	>512(R)	128	256
Imipenem	2(S)	1	1	0.25(S)	1	1	>512(R)	8	>512
Levofloxacin	0.25(S)	–	0.125	0.25(S)	–	0.0625	32(R)	–	4
Meropenem	0.125(S)	0.0313	0.0313	0.125(S)	0.0625	0.0313	64(R)	<1	8
Norfloxacin	2(S)	0.5	1	1(S)	0.5	0.5	32(R)	2	8
Oxacillin	0.25(S)	0.0625	–	0.25(S)	0.125	–	>512(R)	<1	–
Streptomycin	8(R)	4	4	64(R)	32–16	4	128(R)	512	4
Tetracycline	0.5(S)	0.5	0.5	0.25(S)	0.25	0.25	64(R)	16	32
Vancomycin	2(S)	0.5	0.5	2(S)	0.5	0.5	2(S)	2	0.5

### Lactamase activity

**Fig. 1:**
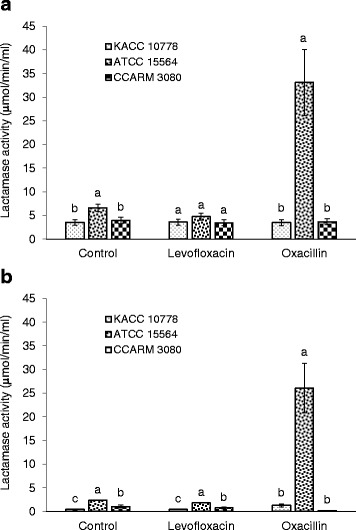
Hydrolyzing activity of extracellular β-lactamase **a** and membrane-bound β-lactamase **b** produced by *Staphylococcus aureus* KACC 10778, *S. aureus* ATCC 15564, and *S. aureus* CCARM 3080 exposed to a half MIC of oxacillin or levofloxacin. Means with different letters (*a*–*c*) on the bars are significantly different at *p* < 0.05

The extracellular and membrane-bound β-lactamase activities were measured in *S. aureus* KACC 10778, *S. aureus* ATCC 15564, and *S. aureus* CCARM 3080 exposed to levofloxacin and oxacillin. No significant change in β-lactamase activities was observed in *S. aureus* KACC 10778 and *S. aureus* CCARM 3080 exposed to levofloxacin. The highest extracellular and membrane-bound β-lactamase activities were observed in *S. aureus* ATCC 15564 exposed to oxacillin, increased to 33 and 26 μmol/min/mL, respectively (Fig. 1).

### Efflux activity

**Fig. 2:**
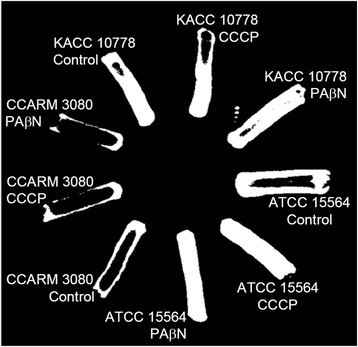
Accumulation and efflux activity of *Staphylococcus aureus* KACC 10778, *S. aureus* ATCC 15564, and *S. aureus* CCARM 3080 on EtBr agar plates containing with and without efflux pump inhibitors (CCCP and PAβN)

**Fig. 3:**
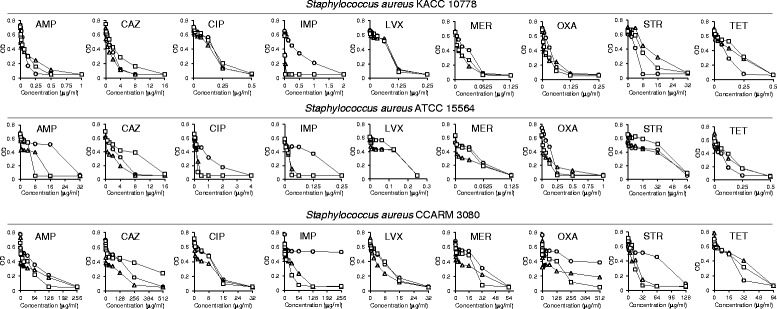
Antibiotic susceptibility of *Staphylococcus aureus* KACC 10778, *S. aureus* ATCC 15564, and *S. aureus* CCARM 3080 in the absent (○) and present of efflux pump inhibitors, CCCP (Δ) and PAβN (□)

The efflux activity of *S. aureus* KACC 10778, *S. aureus* ATCC 15564, and *S. aureus* CCARM 3080 was evaluated on TSA agar plates containing EtBr (Fig. 2). The level of fluorescence intensity was increased in *S. aureus* ATCC 15564 exposed to CCCP and PAβN compared to the control. The highest efflux activity was observed in *S. aureus* CCARM 3080 regardless of the presence of efflux pump inhibitors. The role of efflux pumps in the antibiotic resistance was evaluated in *S. aureus* KACC 10778, *S. aureus* ATCC 15564, and *S. aureus* CCARM 3080 exposed to efflux pump inhibitors, CCCP and PAβN (Fig. 3). The antibiotic susceptibility patterns of all strains exposed to efflux pump inhibitors varied in the types of antibiotics. The antibiotic activity of imipenem against *S. aureus* KACC 10778 was increased in the presence of efflux pump inhibitors, whereas the resistance of *S. aureus* KACC 10778 to streptomycin and tetracycline was increased in the presence of efflux pump inhibitors. The sensitivity of *S. aureus* ATCC 15564 to ampicillin, ciprofloxacin, and imipenem was increased in the presence of CCCP and PAβN. The sensitivity of *S. aureus* CCARM 3080 to imipenem, oxacillin, and streptomycin was increased in the presence of CCCP and PAβN.

### Biofilm-forming ability

**Fig. 4:**
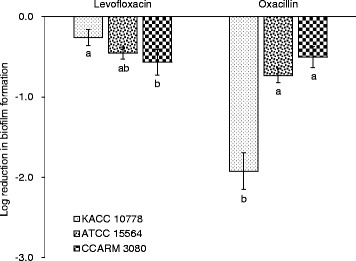
Biofilm-forming ability of *Staphylococcus aureus* grown in a half MIC of oxacillin or levofloxacin. Log reduction was estimated as compared to the control. Means with different letters (*a*–*b*) on the bars are significantly different at *p* < 0.05

The biofilm-forming ability of *S. aureus* KACC 10778, *S. aureus* ATCC 15564, and *S. aureus* CCARM 3080 was evaluated in the presence of levofloxacin and oxacillin (Fig. 4). Compared to the control, the number of biofilm-forming cells of *S. aureus* KACC 10778 was reduced by approximately 2 log CFU/mL in the presence of oxacillin, whereas those of *S. aureus* ATCC 15564 and *S. aureus* CCARM 3080 were reduced by 0.5-1 log CFU/mL in the presence of levofloxacin and oxacillin.

### Differential gene expression

**Fig. 5:**
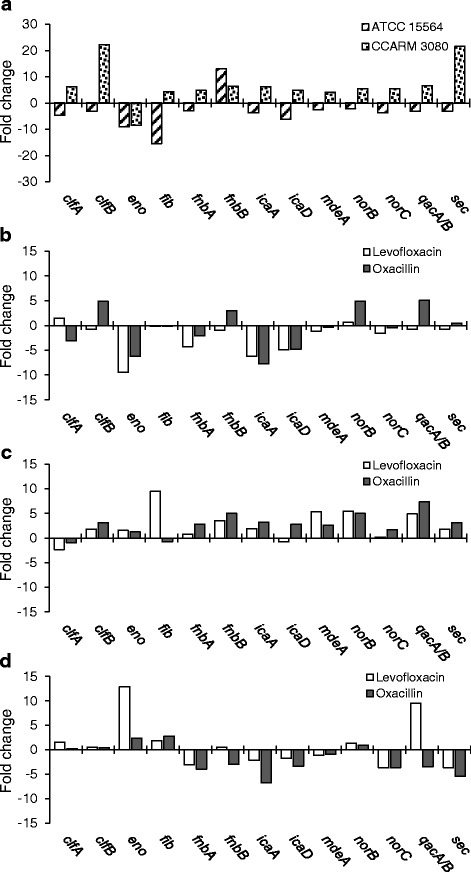
Relative gene expression in antibiotic-resistant strains (*S. aureus* ATCC 15564 and *S. aureus* CCARM 3080) grown in the absence of oxacillin and levofloxacin **a** and *S. aureus* KACC 10778 **b**, *S. aureus* ATCC 15564 **c**, and *S. aureus* CCARM 3080 **d** grown in a half MIC of oxacillin or levofloxacin

The relative expression of adhesion-related genes (*clfA*, *clfB*, *eno*, *fib*, *fnbA*, *fnnB*, and *icaD*), efflux-related genes (*mdeA*, *norB*, *norC*, and *qacA/B*), and enterotoxin gene (*sec*) were observed in *S. aureus* KACC 10778, *S. aureus* ATCC 15564, and *S. aureus* CCARM 3080 grown in the absence and presence of levofloxacin and oxacillin (Fig. 5). The relative expression levels of most selected genes were increased more than 5-fold in antibiotic-resistant *S. aureus* CCARM 3080 (Fig. 5a). The *clfB*, *fnbB*, *norB*, and *qacA/B* genes were overexpressed in *S. aureus* KACC 10778 grown in the presence of oxacillin (>3-fold), whereas the relative expression levels of *eno*, *icaA*, and *icaD* were decreased more than 5-fold in both levofloxacin and oxacillin (Fig. 5b). Most of genes in *S. aureus* ATCC 15564 were slightly overexpressed in the presence of levofloxacin and oxacillin (Fig. 5c). As shown in Fig. 5d, the *eno* and *qacA/B* genes were overexpressed by more than 12- and 9-fold, respectively, in *S. aureus* CCARM 3080 grown in the presence of levofloxacin. The *norB* was slightly overexpressed in *S. aureus* CCARM 3080 grown in the presence of levofloxacin and oxacillin.

## Discussion

This study describes the antibiotic susceptibility and gene expression dynamics of *S. aureus* with different antibiotic resistance profiles when exposed to sub-MICs of levofloxacin and oxacillin. As antibiotic-resistant pathogens are frequently exposed to sublethal concentrations of antibiotic prescribed in hospitals, this study sheds light on the understanding of antibiotic resistance mechanisms and the effectiveness of combination therapy. This study investigated the relationship between phenotypic and genotypic properties of *S. aureus* KACC 10778, *S. aureus* ATCC 15564, and *S. aureus* CCARM 3080 exposed to a half MIC of levofloxacin or oxacillin.

The susceptibilities of *S. aureus* KACC 10778, *S. aureus* ATCC 15564, and *S. aureus* CCARM 3080 to most antibiotics were increased in the presence of levofloxacin and oxacillin, whereas no difference in the susceptibilities of *S. aureus* KACC 10778, *S. aureus* ATCC 15564, and *S. aureus* CCARM 3080 to ceftazidime, chloramphenicol, gentamicin, and vancomycin were observed in the presence of levofloxacin and oxacillin (Table 1). *S. aureus* CCARM 3080 showed the decreased susceptibility to streptomycin. This is in good agreement with a previous report that MRSA exhibited the enhanced resistance to other classes of antibiotics, leading to multidrug resistance [19]. Compared to *S. aureus* KACC 10778 and *S. aureus* ATCC 15564, the decreased oxacillin susceptibility was observed in *S. aureus* CCARM 3080 (MIC > 512 μg/mL), which may be attributed to the activation of penicillin-binding protein (PBP2a) encoded by *mecA*, but not due to the activation of β-lactamase [20, 21]. MRSA can acquire additional resistance to ciprofloxacin, causing a frequent failure in antibiotic treatment [6]. The successive antibiotic treatment may be not effective against bacterial infections because of the increase in bacterial adaptation to initial antibiotic exposure. Thus, the combination therapy is commonly used to broaden antibiotic spectrum and achieve synergistic effect in life-threatening infections [8, 9, 22]. As shown in Table 1, *S. aureus* CCARM 3080 exposed to levofloxacin was more susceptible to most classes of antibiotics than that exposed to oxacillin with the exception of streptomycin and vancomycin. Interestingly, the susceptibility of *S. aureus* CCARM 3080 to streptomycin, however, was decreased in the presence of levofloxacin. The combination therapy can lead to cross-resistance to different classes of antibiotics. The antibiotic susceptibility of *S. aureus* KACC 10778, *S. aureus* ATCC 15564, and *S. aureus* CCARM 3080 exposed to levofloxacin and oxacillin depends on the additional resistance mechanisms. Therefore, the systematic investigation is needed to understand the mechanisms underlying cross-resistance in combination therapy.

The extracellular and membrane-bound β-lactamase activities were influenced by the intrinsic antibiotic resistance of *S. aureus* KACC 10778, *S. aureus* ATCC 15564, and *S. aureus* CCARM 3080 when exposed to levofloxacin and oxacillin. The production of β-lactamase was considerably increased in *S. aureus* ATCC 15564 when exposed to oxacillin, suggesting that the stabilities of β-lactam antibiotics were enhanced against staphylococcal β-lactamases (Fig. 1). Methicillin, oxacillin, cephalothin are less susceptible to hydrolysis by staphylococcal β-lactamases [23]. The resistance of *S. aureus* KACC 10778, *S. aureus* ATCC 15564, and *S. aureus* CCARM 3080 to β-lactam antibiotics may be associated with low-affinity PBPs and membrane permeability [21, 23, 24]. On the other hand, the results imply that the inappropriate selection of antibiotics for combination therapy can lead to the induction of β-lactamases.

*S. aureus* KACC 10778 exhibited low efflux activity in the absence and presence of inhibitors (CCCP and PAβN), whereas the efflux activity of *S. aureus* ATCC 15564 was effectively inhibited by CCCP and PAβN (Fig. 2). The highest efflux activity was observed in *S. aureus* CCARM 3080, which was not even reduced by efflux pump inhibitors. This suggests that there exist the inhibitor-insensitive efflux pump systems in *S. aureus* CCARM 3080, resulting in multidrug resistance. The enhanced efflux activity is a main cause of multidrug resistance in *S. aureus* CCARM 3080 [17].

*S. aureus* KACC 10778, *S. aureus* ATCC 15564, and *S. aureus* CCARM 3080 were exposed to efflux pump inhibitors to characterize the substrate specificity of multidrug efflux pumps (Fig. 3). The MICs of imipenem against *S. aureus* KACC 10778, ampicillin, ciprofloxacin, and imipenem against *S. aureus* ATCC 15564, and imipenem, oxacillin, and streptomycin against *S. aureus* CCARM 3080 were considerably decreased in the presence of efflux pump inhibitors (CCCP and PAβN), suggesting the antibiotic resistance is associated with the proton motive force and substrate competition-dependent efflux systems [25]. The plasma membrane is depolarized in the presence of CCCP, which collapses proton electrochemical gradient [25]. PAβN, a substrate of efflux pumps, acts as an inhibitor competing with antibiotics [14]. The antibiotics inducing PAβN-susceptible efflux can act as potential competitors for multidrug efflux pump systems. The multidrug resistance in bacteria is directly related to the activity of efflux pumps [26, 27]. The reduced susceptibility to the different classes of antibiotics in resistant *S. aureus* CCARM 3080 was due to the interacting resistance mechanisms. As shown in Fig. 3, the MICs of ceftazidime to all strains tests were increased in the presence of PAβN, which corresponds to Table 1 and Fig. 3. The efflux pumps are stimulated by β-lactams. The efflux-mediated antibiotic resistance mechanism can affect the β-lactam uptake, resulting in the increase in β-lactam susceptibility [24]. In contrast, no changes in antibiotic susceptibility between absence and presence of efflux pump inhibitors were observed in *S. aureus* KACC 10778 (ciprofloxacin, levofloxacin, meropenem, and oxacillin), *S. aureus* ATCC 15564 (levofloxacin and meropenem), and *S. aureus* CCARM 3080 (ciprofloxacin and levofloxacin). The observations imply that there are various types of efflux pump systems in *S. aureus* KACC 10778, *S. aureus* ATCC 15564, and *S. aureus* CCARM 3080 [28]. Unlike PAβN, CCCP can decrease the accumulation of antibiotics, resulting in the elevated MICs in *S. aureus* KACC 10778 (ampicillin and streptomycin), *S. aureus* ATCC 15564 (ampicillin and oxacillin), and *S. aureus* CCARM 3080 (ampicillin, imipenem, meropenem, and oxacillin) [28].

The reduction in the number of biofilm cells was more noticeable in *S. aureus* KACC 10778 exposed to oxacillin than the multiple antibiotic-resistant *S. aureus* ATCC 15564 and *S. aureus* CCARM 3080 (Fig. 4). The antibiotic-resistant strains are more likely to obtain cross-protection against various stresses such as acid, heat, and antibiotics [29]. The exposure to certain antibiotics can positively associated with the formation of bacterial biofilms [30]. The degree of biofilm formation depends on the type of antibiotics, including biofilm-inducing (ampicillin, vancomycin, and ceftizoxime) and biofilm-noninducing antibiotics (gentamycin) [30]. The enhanced resistance of biofilms to environmental stresses and antibiotics play an important role in the pathogenesis of bacterial infections [31]. Therefore, the combination therapy needs to take into account the risk factors for multiple antibiotic-resistant bacterial infections.

Most of the overexpressed adhesion-, efflux pump-, and enterotoxin genes were observed in *S. aureus* CCARM 3080 compared to *S. aureus* KACC 10778 (Fig. 5a). The increased expression levels of adhesion-related genes in *S. aureus* ATCC 15564 and *S. aureus* CCARM were directly related to the enhanced biofilm-forming ability [32]. The genes encoding clumping factor, laminin-, and fibronectin-binding proteins were overexpressed in *S. aureus* ATCC 15564 and *S. aureus* CCARM when exposed to levofloxacin and oxacillin, suggesting sublethal concentrations of antibiotics can improve the surface adhesion properties of bacteria [1]. The increased resistance of *S. aureus* CCARM 3080 to multiple antibiotics may be mediated by the overexpression of *mdeA*, *norB*, *norC*, and *qacA/B* genes [33–36]. The expression level of *sec* gene was increased more than 20-fold in *S. aureus* CCARM 3080, suggesting that staphylococcal enterotoxin can be a major cause of staphylococcal infections [37]. Most genes were overexpressed in resistance strains (*S. aureus* ATCC 15564 and *S. aureus* CCARM) when exposed to levofloxacin and oxacillin (Fig. 5c-d). The multidrug resistance of *S. aureus* ATCC 15564 and *S. aureus* CCARM was attributed to the overexpression of efflux pump-related genes (*mdeA*, *norB*, *norC*, and *qacA/B*) [17]. However, the different expression levels of genes encoding efflux pump system in *S. aureus* KACC 10778, *S. aureus* ATCC 15564, and *S. aureus* CCARM 3080 were observed between exposures to levofloxacin and oxacillin. The expression of efflux pump-related genes is induced depending on the exposure to different classes of antibiotics as substrates. Ciprofloxacin is a common substrate of efflux pump systems (NorA, NorB, and NorC) and cationic lipophilic drugs are preferential substrates of QacA/B, a major facilitator superfamily (MFS) [38, 39]. MRSA carries *qacA* and *qacB* in higher rate than MSSA [36]. Kanamycin, linezolid, and lincomycin are the substrates of LmrS multidrug efflux pump, which is similar to EmrB of *Escherichia coli* and FarB of *Neisseria gonorrhoeae* [7]. The major substrate of MdeA multidrug efflux pump is norfloxacin [39]. Tetracycline, fluoroquinolone, and macrolides are the substrates of TetA(K), SdrM, and Mdf(A) efflux pump systems, respectively [39, 40]. The overexpression of the efflux pump systems contributes to the enhanced resistance to their substrates. Bacteria exposed to sublethal concentration can become resistant to multiple antibiotics in a high frequency, whereas lethal concentrations can induce single mutation [1]. Therefore, a proper combination therapy needs to be designed on the basis of antibiotic resistance mechanisms and profiles of target pathogens, which can increase the susceptibility to combination therapy and prevent the spread of newly acquired antibiotic resistance.

## Conclusions

This study highlights the varied and interactive characteristics of antibiotic resistance in *S. aureus* KACC 10778, *S. aureus* ATCC 15564, and *S. aureus* CCARM 3080 exposed to sub-MICs of levofloxacin and oxacillin. The phenotypic and genotypic properties of *S. aureus* with different antibiotic resistance profiles varied in terms of lactamase activity, efflux activity, biofilm-forming ability, and gene expression pattern. The antibiotic-resistant *S. aureus* can acquire cross resistance between different classes of antibiotics when exposed to sublethal concentration, ascribed to differential activity of β-lactamase and efflux pump systems. The results obtained in this study indicate that monitoring antibiotic susceptibility patterns is essential to effectively treat antibiotic-resistant bacterial infections in case of continuous antibiotic exposure, and appropriate combination therapy is required in treating pathogens with different levels of antibiotic resistance. In addition, the characteristic changes in phenotypic and genotypic expression can be used to accurately detect antibiotic-resistant bacteria, which plays an important role in designing antibiotic treatment plan and developing new diagnostic technique for antibiotic resistance. Further systematic studies taking into account molecular approaches are needed to demonstrate the multiple antibiotic resistance mechanisms of pathogens exposed to various antibiotics through combination therapy.

## Abbreviations

CCCP, carbonyl cyanide-*m*-chlorophenyl hydrazine; CFU, colony forming unit; CRE, carbapenem-resistant Enterobacteriaceae; EPI, efflux pump inhibitor; MDR, multidrug resistance; MIC, minimum inhibitory concentration; MRSA, methicillin-resistant *Staphylococcus aureus*; PAβN, phenylalanine-arginine-β-naphthylamide

## Additional files

Additional file 1: Table S1. Description of antibiotics used in this study. (DOCX 13 kb) Additional file 2: Table S2. Primer sequences used in qPCR analysis for *Staphylococcus aureus*. (DOCX 13 kb)
