# Supplementary material for: Phenotypic and genotypic characterisation of multiple antibiotic-resistant Staphylococcus aureus exposed to subinhibitory levels of oxacillin and levofloxacin
Source: BMC Microbiol. 2016 Jul 29;16:170. doi: 10.1186/s12866-016-0791-7 (PMC4966875; doi:10.1186/s12866-016-0791-7)
Supplement: Additional file 1: Table S1. — Description of antibiotics used in this study. (DOCX 13 kb) [file 12866_2016_791_MOESM1_ESM.docx]

**Table S1.** Description of antibiotics used in this study

| Antibiotic  (Abbreviation) | Classification | Inhibitory mechanism | Bacterial susceptibility |
| --- | --- | --- | --- |
| Ampicillin  (AMP) | β-lactam | β-lactamases  Transpeptidase | Broad-spectrum |
| Cefoxitin  (FOX) | β-lactam (Second-generation cephalosporin) | Cell wall synthesis | Broad-spectrum |
| Ceftazidime  (CAZ) | β-lactam (Third-generation cephalosporin) | β-lactamases | Broad-spectrum |
| Ceftriaxone  (CTA) | β-lactam (Third-generation cephalosporin) | Cell wall synthesis  PBP cross-link | Broad-spectrum |
| Chloramphenicol  (CHL) | Amphenicol | Protein synthesis  Peptidyl transferase | Broad-spectrum Bacteriostatic |
| Ciprofloxacin  (CIP) | Second-generation Fluoroquinolone | DNA gyrase  Topoisomerase IV | Broad-spectrum |
| Gentamicin  (GEN) | Aminoglycoside | Protein synthesis  30S subunit | Broad-spectrum |
| Imipenem  (IMP) | β-lactam (Carbapenem) | Cell wall synthesis | Broad-spectrum |
| Levofloxacin  (LVX) | Fluoroquinolone | DNA gyrase  Topoisomerase IV | Broad-spectrum |
| Meropenem  (MER) | β-lactam (Carbapenem) | β-lactamase | Broad-spectrum  Bactericidal |
| Norfloxacin  (NOR) | Fluoroquinolone | DNA gyrase  Topoisomerase II and IV | Broad-spectrum |
| Oxacillin  (OXA) | β-lactam | β-lactamase | Narrow-spectrum |
| Streptomycin  (STR) | Aminoglycoside | Protein synthesis  30S subunit | Broad-spectrum |
| Tetracycline  (TET) | Tetracycline | 30S subunit | Broad-spectrum |
| Vancomycin  (VAN) | Glycopeptide | Cell wall synthesis | Narrow-spectrum |
