# Supplementary material for: Phenotypic and genotypic characterisation of multiple antibiotic-resistant Staphylococcus aureus exposed to subinhibitory levels of oxacillin and levofloxacin
Source: BMC Microbiol. 2016 Jul 29;16:170. doi: 10.1186/s12866-016-0791-7 (PMC4966875; doi:10.1186/s12866-016-0791-7)
Supplement: Additional file 2: Table S2. — Primer sequences used in qPCR analysis for Staphylococcus aureus. (DOCX 13 kb) [file 12866_2016_791_MOESM2_ESM.docx]

**Table S2.** Primer sequences used in qPCR analysis for *Staphylococcus* *aureus*

| Gene | Molecular function | Primer name and sequence^*^ |
| --- | --- | --- |
| 16S rRNA | Reference gene | F: CATGCTGATCTACGATTACT  R: CCATAAAGTTGTTCTCAGTT |
| *clfA* | Clumping factor | F: ATTGGCGTGGCTTCAGTGCT  R: CGTTTCTTCCGTAGTTGCATTTG |
| *clfB* | Clumping factor | F: ACATCAGTAATAGTAGGGGGCAAC  R: TTCGCACTGTTTGTGTTTGCAC |
| *eno* | Laminin binding protein | F: ACGTGCAGCAGCTGACT  R: CAACAGCATYCTTCAGTACCTTC |
| *fib* | Fibronectin binding protein | F: CTACAACTACAATTGCCGTCAACAG  R: GCTCTTGTAAGACCATTTTCTTCAC |
| *fnbA* | Fibronectin binding protein | F: CATAAATTGGGAGCAGCATCA  R: ATCAGCAGCTGAATTCCCATT |
| *fnbB* | Fibronectin binding protein | F: GTAACAGCTAATGGTCGAATTGATACT  R: CAAGTTCGATAGGAGTACTATGTTC |
| *icaA* | Intercellular adhesion protein | F: CCTAACTAACGAAAGGTAG  R: AAGATATAGCGATAAGTGC |
| *icaD* | Intercellular adhesion protein | F: AAACGTAAGAGAGGTGG  R: GGCAATATGATCAAGATAC |
| *mdeA* | Multidrug efflux | F: GTTTATGCGATTCGAATGGTTGGT  R: AATTAATGCAGCTGTTCCGATAGA |
| *norB* | Efflux transporter protein | F: AGCGCGTTGTCTATCTTTCC  R: GCAGGTGGTCTTGCTGATAA |
| *norC* | Efflux transporter protein | F: AATGGGTTCTAAGCGACCAA  R: ATACCTGAAGCAACGCCAAC |
| *qacA/B* | Antiseptic resistance protein | F: GCTGCATTTATGACAATGTTTG  R: AATCCCACCTACTAAAGCAG |
| *sec* | Enterotoxin | F: TGTACTTRTAAGAGTTTATGAAAATA  R: TCCTAGCTTTTATGTCTAGTTCTTGAG |

* F, forward; R, reverse.
